# Reentry: a key mechanism for integration of brain function

**DOI:** 10.3389/fnint.2013.00063

**Published:** 2013-08-27

**Authors:** Gerald M. Edelman, Joseph A. Gally

**Affiliations:** The Neurosciences Institute, La Jolla, CA, USA

**Keywords:** reentry, white matter, corticocortical networks, fiber tracts, consciousness, brain function

## Abstract

Reentry in nervous systems is the ongoing bidirectional exchange of signals along reciprocal axonal fibers linking two or more brain areas. The hypothesis that reentrant signaling serves as a general mechanism to couple the functioning of multiple areas of the cerebral cortex and thalamus was first proposed in 1977 and 1978 (Edelman, 1978). A review of the amount and diversity of supporting experimental evidence accumulated since then suggests that reentry is among the most important integrative mechanisms in vertebrate brains (Edelman, 1993). Moreover, these data prompt testable hypotheses regarding mechanisms that favor the development and evolution of reentrant neural architectures.

## INTRODUCTION

A large and diverse body of evidence suggests that intermittent signaling along reentrant paths is critical to a variety of neural functions in vertebrate brains, ranging from perceptual categorization to motor coordination. Reentry takes on a variety of forms enabling many different processes. These processes facilitate the coordination of neuronal firing in anatomically and functionally segregated cortical areas. By these means they bind cross-modal sensory features by synchronizing and integrating patterns of neural activity in different brain regions. By sustaining attention and short-term memory, reentry might even play a central role in generating conscious awareness. Reentrant signaling is a ubiquitous and dominant structural and functional motif of vertebrate telencephalons (Edelman, 1989). Reentry has, however, rarely, if ever, been characterized in an invertebrate nervous system, and it may be a relatively recent evolutionary innovation.

Before considering the diverse experimental observations suggesting the operation of neural reentry, clarification of nomenclature will be useful. It is important, for example, to distinguish between the terms reentry and feedback. Feedback, as originally defined and used in control system theory (Wiener, 1948), refers to a process in which a signal whose magnitude is related to the difference between desired and actual output is transmitted along a pre-specified path for error correction and control. Error signals travel along a single path different from the forward path to avoid ambiguity among choices of error correction. In contrast, neural reentry does not utilize fixed error-correcting functions or paths. It occurs in selectional systems across multiple reciprocal paths. Moreover, the significance of neural signaling is not pre-specified or determined *a priori*, but rather is acquired through experience. Reentrant neural processes involve the simultaneous exchange of signals in a coordinated manner among multiple dispersed neuronal populations.

**FIGURE 1:**
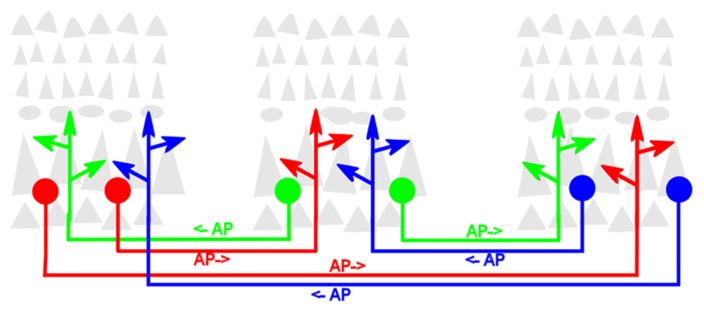
**Schematic diagram of reentrant white-matter fiber bundles linking distant cortical areas.** Gray triangles represent pyramidal neurons that comprise the bulk of neocortical gray matter. For clarity, the dense packing of axonal and dendritic arbors that link neurons within the cortical gray matter is not shown. Colored filled circles represent excitatory neurons projecting axons that send reentrant action potentials bidirectionally between areas. Colored arrowheads represent presynaptic terminals of these axons. AP→ indicates direction of signaling.

A second definitional point is largely a matter of taste. While from its inception as a cortical process (Edelman, 1978) reentry was used as the descriptor, other authors have since used different words, such as “recurrent” (Lamme and Roelfsema, 2000), “recursive” (Pollen, 2003) or “top-down” and “bottom-up” (Van Essen, 2005) to refer to the same process. The context usually affords no ambiguity. However, for these various terms to retain their maximal utility, it is important that each maintains or enhances the important distinction it intends or implies. In this review, reentrant processes will be more precisely defined as those that involve one localized population of excitatory (i.e., glutamatergic) neurons simultaneously both stimulating, and being stimulated by, another such population. The structural architecture that generates this process is likewise referred to as reentrant. The process does not suggest or require that any individual neuron in one population be reciprocally synaptically linked to any specific neuron in the second population. The diagram in **Figure 1** illustrates reentrant connections linking neuronal populations that constitute the gray matter in dispersed cortical areas.

## FUNCTIONS OF REENTRY

An increasing number of investigators of the varied functions mediated by the cerebral cortex have invoked various roles for reentry. In this section we consider experimental findings that illustrate the multiplicity and diversity of functions in which reentrant signaling has been proposed to play a necessary role. Our aim is to marshal evidence and arguments for the widespread occurrence of reentry in vertebrate brains. We expand briefly on some of these roles and consider studies that provide strong empirical evidence for the existence of these proposed functions. The investigations we cite are representative, and they are by no means exhaustive. The majority, but not all, of these studies focus on reentrant signaling within the cerebral cortex.

Structurally, the cerebral cortex has been shown to develop as a mosaic of anatomically and functionally segregated areas (Felleman and Van Essen, 1991; Somogyi et al., 1998). Neurons responsive to different features of unimodal or multimodal sensory input distribute themselves into separate cortical areas. Within each area, neurons within the various cortical layers synaptically interconnect to one another to form the dense columnar arrays that constitute gray matter (shown schematically in **Figure 1**). Neurons belonging to different cortical areas are also reciprocally interconnected by reentrant networks of excitatory axons (Markov et al., 2012). These long-range axons fasciculate to form the long fiber bundles that comprise much of the brain white matter (Wedeen et al., 2012).

Each cortical area is also reentrantly interconnected by large numbers of axons to one or more nuclei of the thalamus (Jones, 1985). Evidence suggests that these thalamocortical and corticothalamic connections serve to modulate global levels of brain arousal (Boly et al., 2012). They also help to determine which of the complex and dynamic patterns of environmental signals arriving in the thalamus from sense organs will be relayed on to the cortex (Bollimunta et al., 2011).

Reentrant neuronal circuits self-organize early during the embryonic development of vertebrate brains (Singer, 1990; Shatz, 1992). It is thought that the spontaneous as well as stimulus evoked patterns of spiking activity among newly generated cerebral neurons help to shape the patterns of synaptic connectivity within and among these neuronal populations. Evidence suggests that the pattern of synaptic links within each cortical neighborhood gives rise to populations of locally highly interconnected neuronal groups (Perin et al., 2011). These large repertoires of variant groups include structurally different subgroups that none the less function similarly, i.e., they are degenerant (Edelman and Gally, 2001). By these means, the particular pattern of connectivity within and among neuronal groups is both dependent upon, and determines, the reentrant pattern of spiking activity within developing neural networks. In later portions of this review we consider hypothetical mechanisms that may guide the development of this anatomy.

It has been shown that reentrant cortical networks can give rise to patterns of activity with winner-take-all properties (Douglas and Martin, 2004; Rutishauser and Douglas, 2009). By utilizing local or long-range inhibitory connections as well as excitatory reentrant connections, the distributed pattern of reentrant activity in which the brain is engaged at any one instant can act to inhibit, suppress, or compete with conflicting alternative response patterns. In this manner reentry can contribute to the unity and temporal continuity of brain function.

The reentrant architecture of vertebrate brains can also generate spontaneous rhythmic activity (Sanchez-Vives and McCormick, 2000; Tahvildari et al., 2012). By its very nature the mutual exchange of action potentials transmitted via reciprocal paths generates oscillatory behavior such as that observed in electrical signals recorded from functioning brains. Neuronal excitability, anatomic connectivity, and patterns of synaptic efficacies appear to self-organize to give rise to widely distributed dynamic patterns of activity within subsets of cortical and subcortical structures. In living brains the levels of activity measured in reentrantly connected functional networks fluctuate in a coordinated fashion (Friedman-Hill et al., 2000; Shu et al., 2003). Such temporally varying patterns of synchronous activity arising among reentrantly linked groups of cerebral neurons can serve to correlate and bind together features present in sensory input signals or in cortically constructed imagery.

It has been shown that intrinsic properties of reentrant neural anatomy and physiology can also make possible temporally sustained patterns of spiking activity (Chawla et al., 2001; Gollo et al., 2010). These, in turn, may provide the necessary substrates for the temporally modulated control of system output. This capability could in principle contribute to the ability of the cortex to execute timed, sequential, or willed processes, such as maintaining working memory, manipulating mental constructs, or issuing segmented motor commands (Georgopoulos and Stefanis, 2007).

The patterns and magnitudes of brain blood oxygen levels measured in the various anatomic components of living brains fluctuate every few seconds in response to variations in local neural activity. Brain imaging techniques reveal correlations in the timing of these fluctuations among networks of anatomically dispersed but functionally related cortical areas (Fox and Raichle, 2007). These global patterns of functional connectivity are likely to be maintained via long-range reentrant axonal fibers that link nodes of these networks. Reentrant neural activity is also thought to underlie the oscillating electrostatic fields generated within and among such networks. The large-scale synchronization observed in these signals in magnetoencephalography (MEG) and electroencephalography (EEG) studies of functioning human brains has been proposed to play a role in coherent behavior, cognition, and perception (Varela et al., 2001; Hipp et al., 2011).

It has been proposed that phenomenal experience itself is entailed by appropriate reentrant intracortical activity (Edelman, 1993, 2004; Edelman and Tononi, 1999). Evidence suggests that synchronous exchanges of signals among neuronal groups in dispersed cortical areas correlate with, and bind together, the multiple but distinguishable features of unified, conscious scenes. Moreover, activity dependent alterations in neuronal or synaptic properties over time can give rise to temporally alternating percepts in response to constant sensory input, e.g., binocular rivalry or ambiguous figures (Doesburg et al., 2009; Edelman et al., 2011; Buzsáki and Wang, 2012). Reentry may thus be critical for transformation of sensory neural activity into a stable, consciously reportable percept.

As mentioned above, extensive reentrant connectivity links all cortical areas to the thalamus. An important physiologic role for thalamic structures is revealed by the anesthetic effects of propofol. This anesthetic agent has been shown to act to induce a high degree of synchronicity in slow oscillations of electrostatic potentials that are known to be mediated by thalamocortical reentry. It has been argued that this widespread synchronicity precludes the differentiated functioning within and among different brain areas that underlies those mental discriminations constituting consciousness (Ching et al., 2010; Boly et al., 2012; Kim et al., 2012).

Attention, the property that, at any one time, brains direct and focus resources onto a very small fraction of possible tasks or topics available to it, might arise in part as a consequence of the winner-take-all aspect of reentrant circuits. The content of the focus of attention may be determined either by the intrinsic character of the sensory input or by endogenous networks of distributed neural activity that entail conscious thought or awareness. The ability of reentry to direct or modulate competition for causal efficacy among interconnected neuronal groups may facilitate selective and deliberate attention to sensory input or to other mental content (Fuster, 2000; Hamker, 2005; Gazzaley and Nobre, 2012).

We end this brief survey by pointing out that the selective advantage conferred on an animal by its brain is only made evident by adaptation in its motor output. The complex pattern of muscular contractions that underlie primate behavior, for example, is controlled by the combined output of at least six cortical areas in the frontal lobe. Besides being reentrantly linked to one another and to other, non-motor cortical areas, each of these motor areas projects directly to the spinal cord (Burnod et al., 1999).

## ANATOMICAL SUBSTRATES OF REENTRY

Much remains to be learned about the anatomy, evolution and development of the anatomic substrates that generate and transmit reentrant signals. Reentry occurs within local as well as long-range neuronal networks. We consider each in turn.

Locally within the upper layers of neocortical gray matter some pyramidal neurons project axons horizontally for up to several millimeters. The size, shape, location, and connectivity of the axonal arbors of these corticocortical neurons have been found to be modulated by patterns of spiking activity in accord with a “fire-together, wire-together” rubric. The operation of this rule in the context of normal sensory input automatically segregates cortical areas into local “patches.” Neurons within any particular patch respond to specific stimuli with patterns of spiking activity that are unlike those occurring in adjacent cortical cells but similar to those observed in patches that self-organize at other cortical locations. It has been demonstrated that dispersed neuronal components within these so-called “superficial patch systems” (Moeller et al., 2008; Muir et al., 2011) respond to specific stimuli as reentrantly interconnected neuronal groups.

Neural signaling among brain areas separated by longer distances is largely mediated by networks of long-range tracts of myelinated axons that constitute white matter. Our understanding of the detailed anatomic organization of white matter tracts in growing and functioning human brains is presently flourishing as a result of recent and rapid improvements in diffusion magnetic resonance imaging (MRI) methodology (Assaf and Pasternak, 2008). Bundles of reentrant axons in white matter constitute three anatomically and functionally distinct categories of pathways: transcallosal, corticothalamic, and associational. (1) Transcallosal fibers project from the cortex of one cerebral hemisphere through the corpus callosum and reenter the cortex at a symmetrical location in the opposite hemisphere. (2) Corticothalamic axons project from each specific neocortical location to innervate topographically the thalamic nucleus from which the corresponding thalamocortical axons arise. (3) Each long-range associational fiber specifically interconnects two different cortical areas within a single hemisphere. Throughout much of the human cerebral white matter, fiber bundles within any one of these three categories lie parallel to one another. The bundles in any one category tend to lie perpendicular to those in the other two categories (Wedeen et al., 2012); i.e., thalamocortical axons tend to project perpendicular to cortical surface, transcallosal (associational) fibers project tangentially in a mediolateral (frontoposterior) orientation.

It is useful to contrast the different patterns of growth and functioning of these three fiber types. The development and functioning of transcallosal axonal links seems relatively straightforward. Axons grow in symmetrical morphogen gradients to innervate the cortical areas serving similar functions in the opposite hemisphere in a manner that acts to integrate overall organismal behavior. Perhaps the most remarkable aspect of these fibers is their dispensability: humans and other animals lacking an intact callosum, either as a result of a genetic abnormality or surgery, show a nearly normal phenotype (Kamnasaran, 2005).

Intact development and functioning of corticothalamic connectivity, on the other hand, is crucial for animal survival. In addition to serving as a gate of most sensory input into the telencephalon, these pathways play critical roles in regulating arousal states and in directing and moderating attention to determine mental contents and behavioral output. It has been suggested that the conduction velocities of axons within this system adapt in a manner that allows synchrony of neuronal activity throughout the brain, even at anatomically widely separated cortical areas (Chawla et al., 2001; Gollo et al., 2010).

The functional importance of corticocortical associational fiber tracts is well documented and appreciated. These serve the so-called “top-down” and “bottom-up” signaling functions by which sensory input into posterior areas of the brain can be attended to, mentally manipulated, and employed to generate those motor commands that determine animal behavior. It is chiefly this network of connections that integrates memories, present input, and imagined futures in the process of creating a conscious scene (Edelman, 1993; Fuster, 2008). Association fibers are predominantly composed of axons of a specific subpopulation of early differentiating glutamatergic neurons. These neurons arise in deep cortical layers and can be identified by their distinctive morphology and antigenic markers (Arimatsu et al., 2003). However, neither the presynaptic inputs nor the postsynaptic targets of these neurons have so far been described in sufficient detail.

Although it appears that every cortical area is connected to at least one other area in the associational network, this connectivity is very sparse. Since axons within white matter tracts imaged do not appear to branch, those within any one fiber bundle are assumed to link two separate volumes of target gray matter (**Figure 1**). MRI techniques do not reveal whether axons within associational tracts project in the same or in opposing directions. In those few instances in which the issue has been experimentally investigated using retrograde tracing methods, the evidence suggests that most, but not all, of these white matter associational tracts are indeed bidirectional (Markov et al., 2012). Clearly, such an anatomic arrangement would facilitate reentrant activity in a functioning brain.

## DEVELOPMENT AND EVOLUTION: AN HYPOTHESIS

Available empirical evidence prompts us to speculate that the evolution and developmental functioning of the distinctive mechanisms that give rise to associational white matter tracts played a central role in the evolution of vertebrate brains. With this in mind, we propose a hypothetical developmental mechanism to account for the formation of these tracts.

In this proposal, clusters of a subpopulation of neurons differentiating in deep, early-appearing cortical layers of the developing embryonic cerebrum extend axons that bundle together to form fasciculated tracts. These tracts grow beneath the developing cortical layers prior to, and thus independent of, patterned sensory input relayed by the thalamus. Since neighboring neurons would tend to project axons that bundle together, this provides a mechanism whereby topographic order can form and be maintained among axons within white matter tracts. Evidence that this occurs has been reported in studies of the growth of thalamocortical axons into early visual cortex (Lokmane et al., 2013). This mechanism would facilitate the development of similar topographic order in reentrantly interconnected cortocal areas in a manner not directly dependent on spatiotemporal patterns of spiking activity.

It is proposed that tracts of pioneering corticocortical axons are guided by the local extracellular cues to grow subcortically along self-organizing networks of fiber bundles. Within the developing white matter, according to this account, they encounter, and then fasciculate with and grow upon, similar bundles of axons growing in the opposite direction. Each axon would then continue to extend along an antiparallel substrate until its growth cone reenters the developing cortical layers at the site at which its guiding substrate originated. Within this gray matter environment the axonal bundles dissociate and individual axons for the first time encounter dendritic sites that provide potential postsynaptic targets.

The hypothetical process described above would repeat and continue until every cortical area projects axons in white matter that bundle together with axons that guide them to a second localized cortical area. As a consequence of this developmental process, each associational tract would be structured to send action potentials bidirectionally between two separate but reentrantly interconnected cortical areas. This network of white matter fiber bundles initially organized among axons of early forming deep-layer cortical neurons could also serve as a scaffold to guide the growth of shorter-range axons of upper-layer neurons that differentiate subsequently. This might also contribute to the reentrant white matter connections that link neuronal groups located in superficial cortical layers

What would be the nature of the signals sent along the axons in these reentrant fiber bundles and how might they contribute to brain function? The timing and rate of action potentials sent in each direction would be determined by the pattern and level of neuronal activity in the cortical volumes that include the cell body and dendrites of the projecting neurons. Each such volume would overlap with the volume containing the terminal arbors of the reentrant axons. If the presynaptic sites within these arbors connected primarily to local inhibitory neurons, a system might arise in which the firing of neurons in one site would transiently hyperpolarize and thus inhibit the firing of neurons in the other site. Thus, this reentrant architecture could give rise to those networks of synchronously oscillating, but anatomically widely distributed cortical areas that are experimentally observed (Siegel et al., 2012).

The hypotheses put forward here regarding the functioning, development, and detailed microanatomy of long range reentrant structures in cerebral white matter make clear-cut predictions that are subject to experimental investigation. Fortunately, impressive advances in techniques that allow the imaging of genetically labeled axonal tracts *in situ* have been reported (Chung et al., 2013). Application of these approaches should either corroborate or modify these hypotheses. In any event, the experimental data summarized here suggest exciting insights into the integration of brain function.

## SUMMARY

A large and diverse body of experimental evidence indicates that processes of reentry play widespread and essential roles in vertebrate brain function, evolution, and development. The reciprocal exchange of signals among neural networks in distributed cortical and corticothalamic areas, when combined with appropriate mechanisms for synaptic plasticity, results in the spatiotemporal integration of patterns of neural network activity. This allows the brain to categorize sensory input, remember and manipulate mental constructs, and generate motor commands. Moreover, these reentrant processes have self-organizational properties that permit robust functioning in the face of genetic or environmentally induced malformation or injury and that allowed for the rapid evolution of the human brain. The use of new anatomical tracing methods to investigate and analyze of these processes in subcortical as well as cortical structures should contribute strongly to our understanding of higher brain function.

## Conflict of Interest Statement

The authors declare that the research was conducted in the absence of any commercial or financial relationships that could be construed as a potential conflict of interest.
